# Chronic recurrent multifocal osteomyelitis in pediatric patients: A Chinese single center observational study and literature review

**DOI:** 10.1097/MD.0000000000040805

**Published:** 2024-12-06

**Authors:** Minhua Hu, Wenxing Zeng, Jingtao Zhang, Hongsong Yan, Feng Huang, Hao Xiong, Bin Fang, Yue Li

**Affiliations:** a Zhongshan Hospital of Traditional Chinese Medicine Affiliated to Guangzhou University of Traditional Chinese Medicine, Zhongshan, China; b Guangzhou University of Chinese Medicine, Guangzhou, China; c The First Affiliated Hospital of Guangzhou University of Chinese Medicine, Guangzhou, China.

**Keywords:** chronic nonbacterial osteomyelitis, chronic recurrent multifocal osteomyelitis, pediatric patients, whole-body MRI

## Abstract

Chronic recurrent multifocal osteomyelitis (CRMO) is a rare autoinflammatory disorder that commonly poses diagnostic challenges due to its atypical symptomatology. This observational study aimed to investigate the clinical features, laboratory test results, imaging features, and treatment strategies for pediatric patients with CRMO. We retrospectively analyzed 7 pediatric patients with CRMO treated at the Department of Pediatric Orthopedics, First Affiliated Hospital of Guangzhou University of Chinese Medicine between January 2018 and February 2022. This study aimed to enhance current understanding of CRMO by exploring in-depth clinical data. The study cohort comprised 5 males and 2 females, aged 3 to 13 years. All patients experienced symptoms for a median duration of 6 months prior to hospitalization, at which point they presented with recurrent pain and various accompanying signs including hypothermia (14.28%), swelling (42.85%), localized skin warmth (42.85%), and plantar pustules (14.28%). The femurs (71.42%) and tibia (71.42%) were frequently affected. Radiographic and computed tomography scans revealed osteosclerosis and osteolytic lesions, while magnetic resonance imaging revealed bone marrow edema. Histological examination of bone biopsies from 4 patients revealed fibrous tissue hyperplasia and lymphocytic and neutrophilic infiltration, despite negative bacterial cultures. Laboratory test results were either normal or slightly elevated. Symptomatic improvement was observed in 6 patients (85.72%) treated with nonsteroidal anti-inflammatory drugs, while 1 patient (14.28%) resistant to nonsteroidal anti-inflammatory drugs responded well to tumor necrosis factor inhibitors. The clinical presentation of CRMO lacks specificity, with unexplained bone pain being the most common symptom. Precise diagnosis and timely intervention depend on a thorough magnetic resonance imaging evaluation for lesion detection, which facilitates CRMO diagnosis. This study offers valuable insights into the clinical manifestations, laboratory findings, imaging features, and treatment strategies of CRMO in pediatric patients.

## 1. Introduction

Chronic nonbacterial osteomyelitis (CNO) is a rare, spontaneous inflammatory disease that primarily affects adolescents and children, with a low incidence of only 4 per 1,000,000 individual cases.^[1]^ Giedion et al first described this “subacute,” “symmetrical” bone lesion in 1972, reporting 4 cases of patients with aseptic osteomyelitis characterized by multiple symmetrically distributed lesions.^[2]^ Subsequently, in 1978, several researchers used the term “chronic recurrent multifocal osteomyelitis (CRMO)” to define osteomyelitis in the context of CNO, characterized by the presence of multiple lesions, symmetrical distribution, and recurrent episodes.^[3]^ Numerous recent studies have reported similar diseases. In 1980, Björkstén et al summarized the histopathological features of 14 CRMO cases, describing the early disease phase as acute inflammation dominated by polymorphonuclear leukocytes, and the later disease phase as characterized by lymphocytic infiltration.^[4]^ In 1988, Brown et al reviewed the radiographs and medical records of 11 patients with CRMO, noting that persistent bone lesions that frequently accompanied this disease.^[5]^ Roderick et al subsequently proposed the Bristol diagnostic criteria to improve diagnostic efficiency, shorten the time to diagnosis, and reduce the requirement for bone biopsies.^[6]^ Oliver et al further conducted an international Delphi survey of 259 pediatric rheumatologists to develop standard classification criteria for CNO and CRMO, identifying 31 candidate items to support diagnosis.^[7]^ These studies provided valuable insights into the diagnosis of CRMO, a condition that is commonly underdiagnosed or misdiagnosed in clinical practice.

CRMO is clinically characterized by the presence of osteolytic or osteosclerotic lesions pervasively affecting numerous bones throughout the anatomical framework, accompanied by pain at the lesion sites.^[8]^ Although CRMO is categorized as a form of chronic osteitis, its inherent self-limiting and cyclic trajectory presents a paradoxical clinical phenomenon in which affected patients experience asymptomatic intervals interspersed with episodes of recurrent bone pain or edema stemming from aseptic inflammatory bone disorders, with the nocturnal exacerbation of symptoms being notably pronounced.^[9]^ Furthermore, some patients may additionally present with extra-articular inflammatory skin manifestations, such as psoriasis, acne, and pustulosis, which are commonly associated with synovitis acne pustulosis hyperostosis osteitis (SAPHO) syndrome.^[10]^ In clinical practice, skeletal pain in CRMO cases is frequently misattributed to common childhood pain, further compounding the diagnostic challenges.^[11]^ Furthermore, the resemblance between CRMO and other conditions, including infectious osteomyelitis, osteoporosis, diffuse idiopathic skeletal hyperostosis, reduced alkaline phosphatase, and bone tumors, can exacerbate the complexity of achieving a precise diagnosis.^[12–14]^ CRMO remains inadequately recognized by clinicians, while its complex etiology and pathogenesis remain elusive.

To address this knowledge gap, we conducted a retrospective study analyzing the clinical characteristics of CRMO using comprehensive medical data obtained from patients, with the aim of enhancing clinicians’ understanding of CRMO. The insights derived from this study will provide valuable information for early recognition, accurate diagnosis, and effective treatment of CRMO, thereby improving patient outcomes.

## 2. Materials and methods

Herein, we retrospectively analyzed the medical records of 7 patients diagnosed with CRMO at the Department of Pediatric Orthopedics, First Affiliated Hospital of Guangzhou University of Traditional Chinese Medicine, from January 2018 to February 2022. Given the absence of universally-recognized diagnostic criteria for CRMO, diagnosis relied on a comprehensive assessment of the patient’s medical history, presenting symptoms, and imaging findings.

The inclusion criteria were as follows: disease duration exceeding 6 weeks; presence of 2 or more osteolytic or sclerotic lesions on imaging; and age range of 1 to 18 years. The exclusion criteria were as follows: individuals with tumors, infections, immunodeficiency diseases, or monogenic autoinflammatory diseases.

This study was approved by the Institutional Review Board of the First Affiliated Hospital of Guangzhou University of Chinese Medicine [No. JY2023-077] on August 8, 2023. According to the local ethics committee regulations, as this study was based on medical records and biological samples obtained from previous clinical diagnoses and treatments, the Institutional Review Board of the First Affiliated Hospital of Guangzhou University of Chinese Medicine waived the requirement for informed consent for secondary data analysis.

### 2.1. Clinical data

Clinical characteristics, including biological sex, age at onset, duration of delay in diagnosis, family history, and clinical symptoms of the patients, were recorded. Laboratory evaluation at admission included a complete blood count, along with assessments of C-reactive protein (CRP), erythrocyte sedimentation rate (ESR), and HLA-B27 antigen positivity. Radiography, computed tomography (CT), and magnetic resonance imaging (MRI) were performed to provide radiological evidence.

Histomorphological findings obtained from bone puncture biopsy were documented. The procedure for bone biopsy was as follows: trained orthopedic surgeons performed bone biopsies under sterile conditions. Local anesthesia or sedation was administered prior to the procedure to ensure patient comfort. Under imaging guidance, a specialized biopsy needle was introduced through the skin and soft tissues into the target bone. The needle was then rotated under the applied pressure to penetrate the bone cortex and reach the lesion. Once the target site was reached, the inner trocar was removed, and a core sample of the bone was extracted for further pathological analysis.

Regarding specimen handling and preservation, all biopsy samples were immediately fixed in formalin. Fixed tissues were then processed and embedded in paraffin for histopathological examination.

The treatment strategy employed for the patients was recorded.

### 2.2. Follow-up duration

The patients were followed up for a minimum of 18 months and a maximum of 36 months, with an average follow-up period of 26 months. Follow-up assessments included routine clinical visits and imaging evaluations, which were necessary to monitor disease progression or resolution.

### 2.3. Statistical analysis

Descriptive analyses were performed on all data. Measurement data that adhered to a non-normal distribution are expressed as the M (Q1, Q3), whereas count data are presented as examples (%).

## 3. Results

### 3.1. Baseline demographic data

This case series including 5 boys and 2 girls, with a median age at presentation of 12 years (range: 3–13). The mean duration from symptom onset to admission was 8.2 months. None of the patients had familial CNO (Table 1).

**Table 1:** Characteristics of patients with chronic recurrent multifocal osteomyelitis (CRMO).

Patient	1	2	3	4	5	6	7
Gender	Male	Male	Female	Male	Male	Male	Female
Age at onset (years)	13	11	3	12	13	13	10
Diagnosis delay (months)	5	7	24	12	2	1.5	6
Family history	–	–	–	–	–	–	–
Clinical manifestations							
Bone pain	+	+	+	+	+	+	+
Fever	-	-	-	+	-	+	-
swelling	-	-	-	+	+	+	+
Increased skin temperature	+	-	-	+	+	-	-
Pustulosis	-	-	+	-	-	-	-
Pain site	Knees	Lumbosacral	Knees and calfs	Shoulder	Knees and ankles	Ankles	Ankles
Lesion skeletons on MRI							
Femur	+	-	+	-	+	+	+
Tibia	+	-	+	-	+	+	+
Fibula	+	-	+	-	+	-	-
Other	-	Sacrum	Pelvis, patela	Clavicle	Taluses calcanes	-	Metatarsals, calcaneus, talus
Treatments	NSAIDs	NSAIDs	NSAIDs	NSAIDs, TNF-α inhibitors	NSAIDs	NSAIDs	NSAIDs

### 3.2. Clinical manifestations

In all cases the children initially presented with localized skeletal pain. The most frequent sites of pain were the knee (observed in 3 cases, 42.85%) and ankle (also noted in 3 cases, 42.85%). Additionally, pain occurred in the lower leg (1 patient, 14.28%), lumbosacral region (1 patient, 14.28%), and foot (1 patient, 14.28%). In addition to localized pain, low-grade fever was observed in 1 of 7 CRMO cases. Swelling at the site of pain was identified in 3 patients (42.85%), while 3 patients (42.85%) displayed elevated skin temperature. Notably, extraosseous skin inflammatory disease, characterized by scattered pustules on the palmar toes, was detected in only 1 patient (14.28 %). All children exhibited involvement of multiple skeletal sites, with the femur and tibia being the most commonly affected bones, presenting with lesions in 5 cases (71.42%), followed by the fibula in 3 cases (42.85%). Involvement of the sacral spine, clavicle, pelvis, and foot bones, was less frequent, with each occurring in 1 patient (14.28%) (Table 1).

### 3.3. Laboratory evaluation

Laboratory assessments revealed a low incidence of inflammatory syndromes in all patients. The leukocyte count in the peripheral blood fell within the normal range (4–10 × 10^9^/L) for all CRMO patients, with the exception of 1 patient who exhibited a heightened serum CRP concentration at 9.88 mg/L and 1 patient with heightened ESR at 22 mm/h. Furthermore, concurrent alterations in the ratio of peripheral blood neutrophils (neutrophil percentage, NEU%) and lymphocyte count (lymphocyte percentage, LEM%) were observed in 3 patients, in which a decrease in NEU% coincided with an increase in LEM%. Additionally, all 6 patients demonstrated an upward trend in peripheral blood platelet count. Notably, Human Leukocyte Antigen B27 testing yielded negative results in all 4 patients who underwent the test, as shown in Table 2.

**Table 2:** Laboratory evaluation and histologic results of bone biopsies.

Case	1	2	3	4	5	6	7
WBC × 10^9^L	6.29	7.56	8.76	6.31	5.71	5.18	6.41
ESR (mm/h)	5	22↑	12	4	5	10	7
CRP (mg/L)	1.15	1.61	9.88↑	1.19	1.41	1.74	3.44
NEU (×10^9^L)	2.43	4.69	4.69	3.9	2.2	2.95	2.68
LEN (×10^9^L)	3.14	2.3	3.28	2.03	2.71	1.72	3.04
NEU%	38.7↓	62	56.6	62	38.5↓	57	41.8↓
LEN%	50.0↑	30.4	37.4	32.1	47.4↑	33.3	47.5↑
NEU%/LEM%	0.77	2.04	1.51	1.92	0.81	1.72	0.88
PLT × 10^9^L	304↑	351↑	360↑	320↑	248	397↑	338↑
HLA-B27	negative	negative	negative	negative	-	-	-
Bone biopsies	+	+	+	+	Not do	Not do	Not do
Bacterial culture	negative	negative	negative	negative	-	-	-
Histologic evaluation							
Fibrous tissue hyperplasia	+	+	+	+	Not do	Not do	Not do
Lymphocytic infiltration	+	+	+	+	Not do	Not do	Not do
Neutrophil infiltration	+	+	-	+	Not do	Not do	Not do

CNO = chronic nonbacterial osteomyelitis, CRP = C-reactive protein, ESR = erythrocyte sedimentation rate, LEM = lymphocyte, NEU = neutrophil, PLT = peripheral blood platelet counts, TNF-α = tumor necrosis factor-alpha.

### 3.4. Imaging features

**Figure 1.**
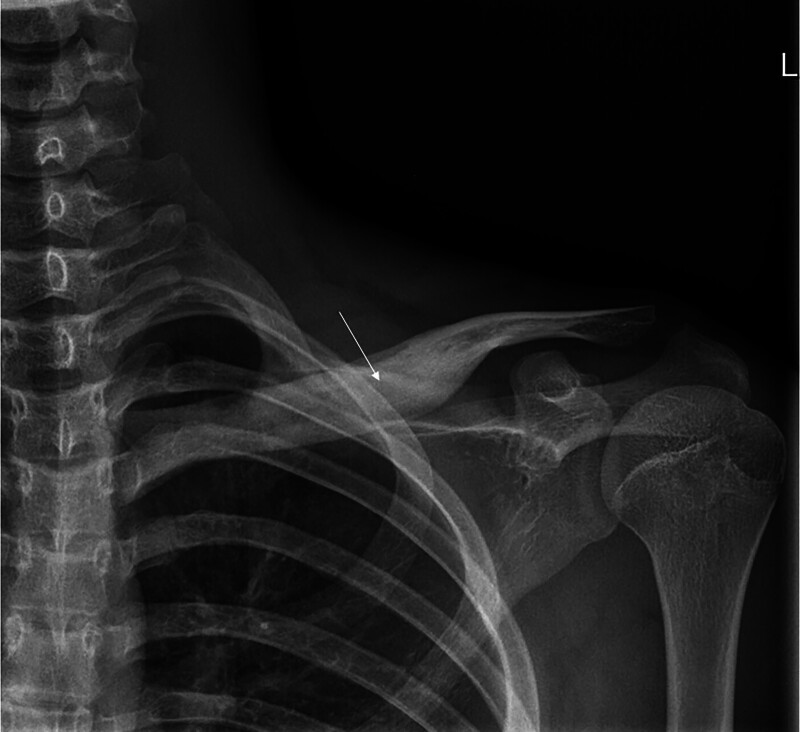
Radiograph showed areas of osteosclerosis (white arrow) on the clavicle of a 12-year-old boy.

**Figure 2.**
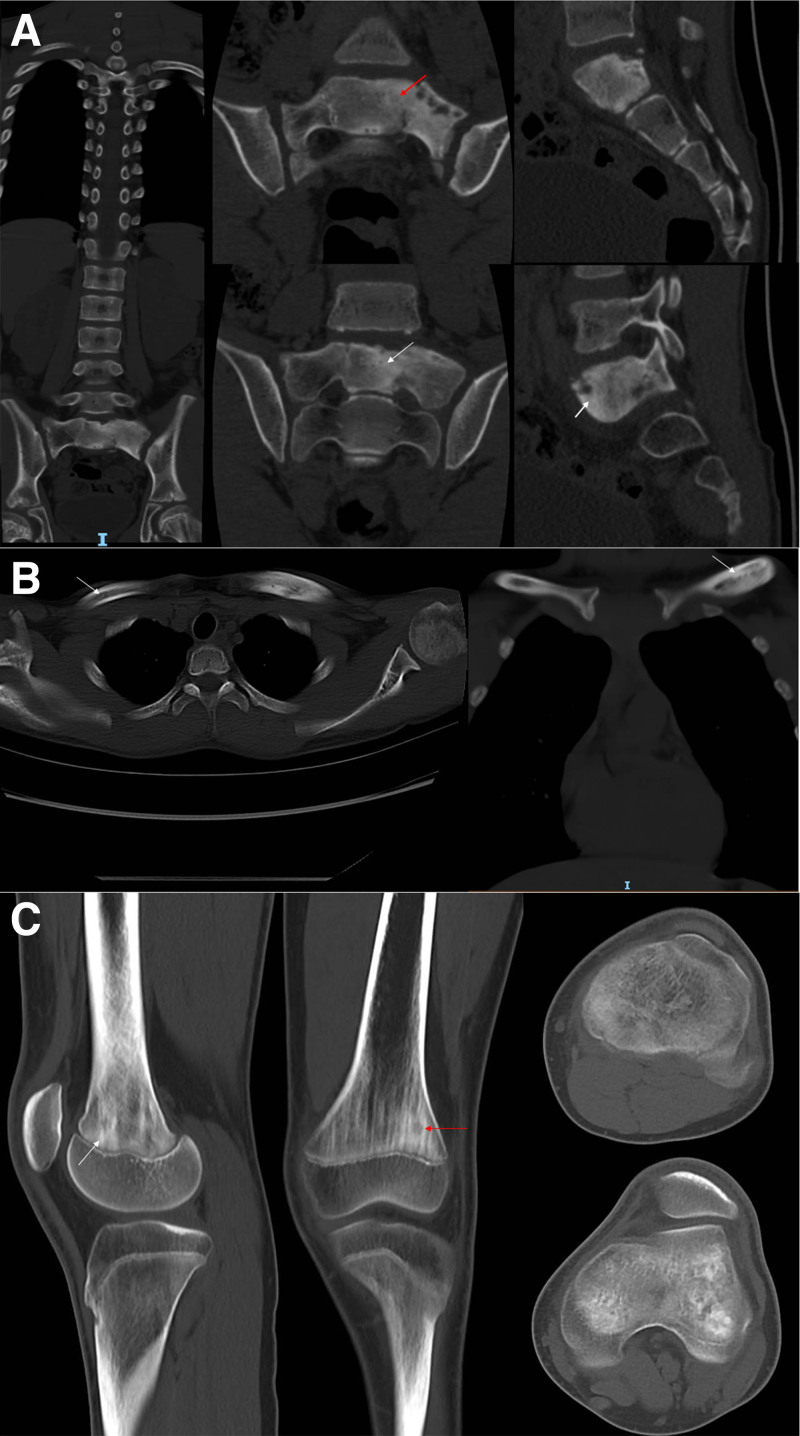
Findings of computed tomography in CRMO patients. (A) Lytic lesion (red arrow) and sclerosis (white arrow) of the sacrum in an 11-year-old boy; (B) intramedullary heterogeneous density (white arrow) of the clavicle in a 12-year-old girl; (C) lytic lesion (red arrow) and sclerosis (white arrow) of the sacrum in a 13-year-old boy. CRMO = chronic recurrent multifocal osteomyelitis.

**Figure 3.**
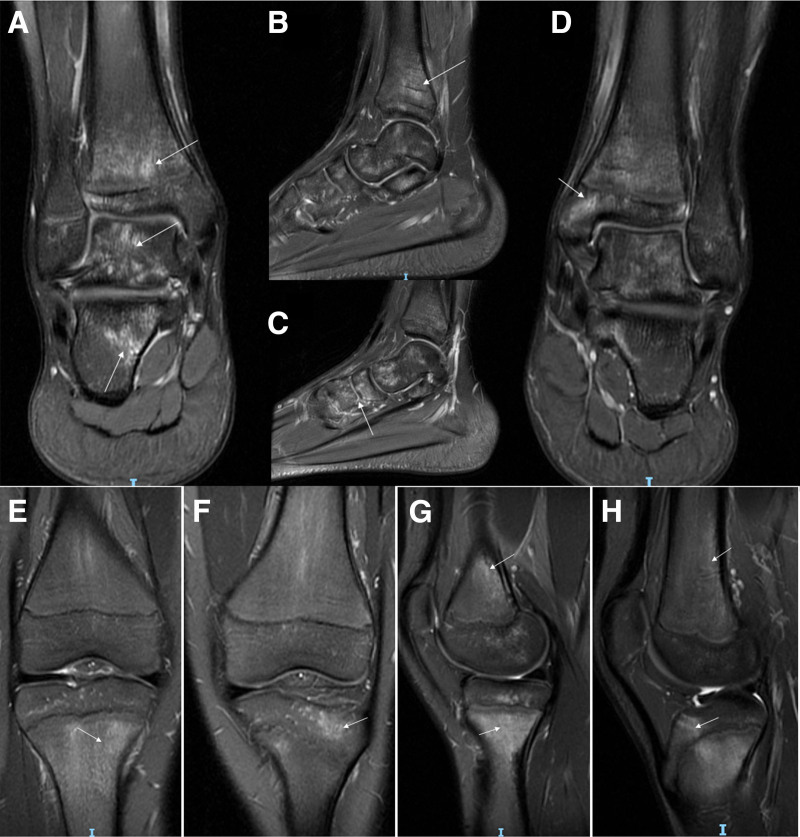
Abnormal signal intensity on MRI of the ankles and knees of a 13-year-old boy. (A and B) The lesions were hyperintense (white arrows) on the coronal and sagittal T2-weighted images of the left ankle; (C and D) the lesions were hypointense on T2-weighted images of the right ankle, involving the tibias, taluses, calcaneus, and dice bones; (E–H) the lesions were hyperintense (white arrows) on the coronal and sagittal T2-weighted images of the right and left knees, involving the femurs and tibias. MRI = magnetic resonance imaging.

**Figure 4.**
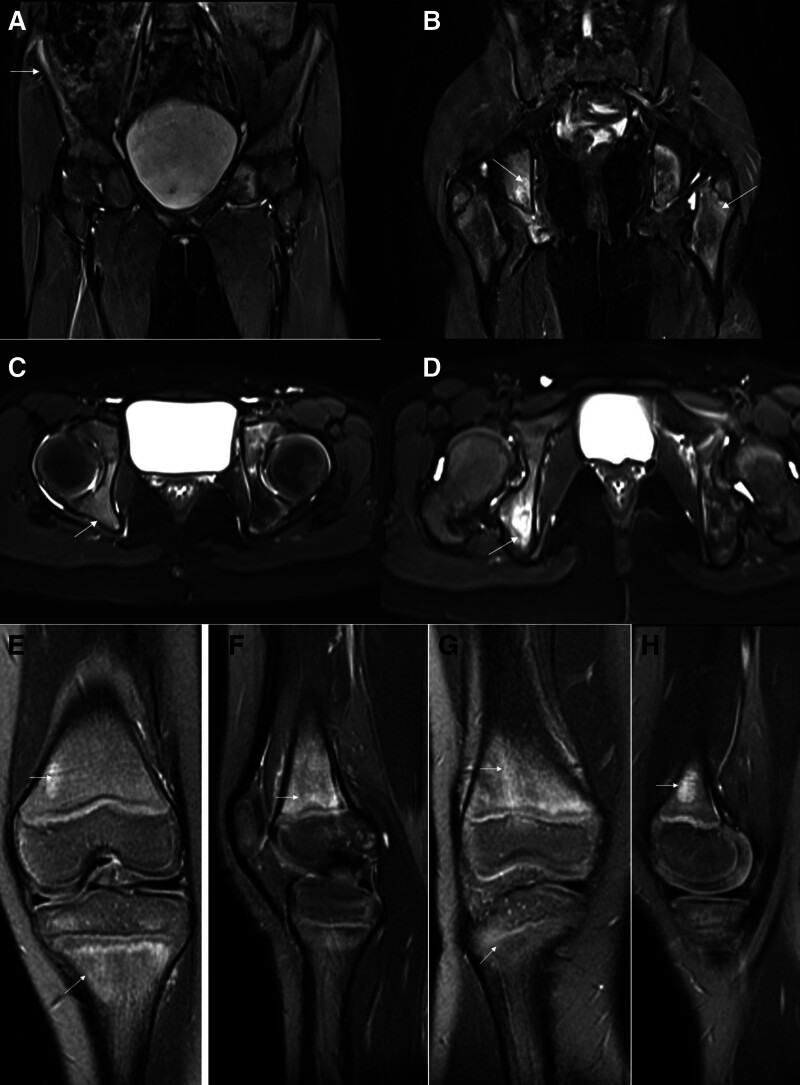
Abnormal signal intensity on MRI of the hips and knees of a 3-year-old girl. (A–D) Hyperintensity (white arrows) on T2-weighted images, involving the pelvis and femur; (E–H) T2-weighted fat-saturated MRI showed metaphyseal and epiphyseal osseous edema of the tibias and femurs (white arrows). MRI = magnetic resonance imaging.

Radiographs were obtained from 6 patients with CRMO, with no evidence of lesions in 3 of them. In the remaining 3 patients, radiography revealed signs of bone destruction, such as osteosclerosis or osteolytic lesions (Fig. 1). CT scans were performed in 3 patients, revealing lytic lesions and sclerosis with hypodense shadowing in the medullary cavity (Fig. 2). MRI was conducted on all 7 patients who exhibited multiple foci of bone marrow edema, accompanied by high signal changes on T2-weighted images (Figs. 3 and 4), predominantly located in the epiphysis of the long bones, with a few in the diaphysis.

### 3.5. Histologic evaluation

**Figure 5.**
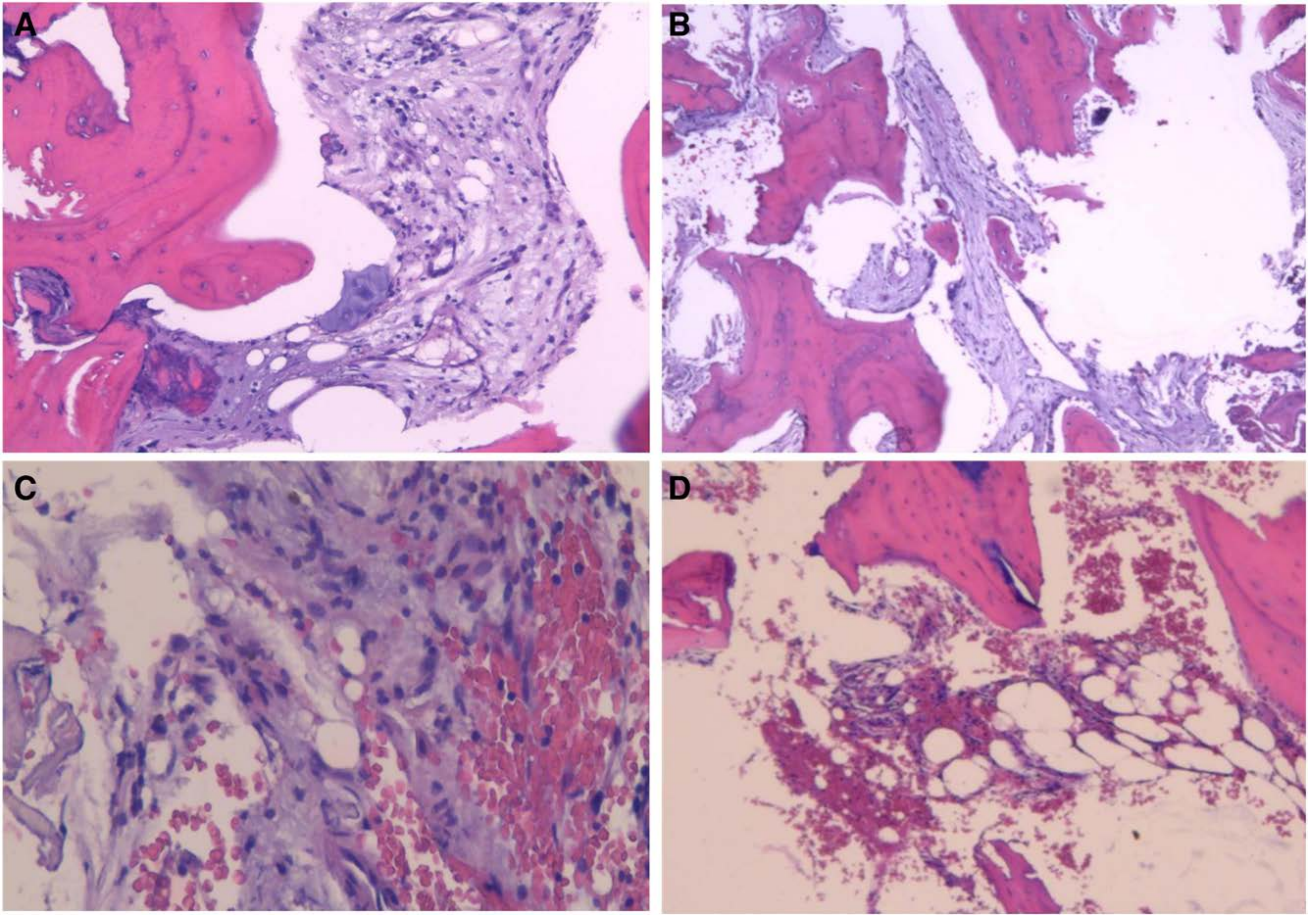
Histopathology of CRMO showed fibrous tissue hyperplasia with lymphocyte and neutrophil infiltration (hematoxylin-eosin staining). CRMO = chronic recurrent multifocal osteomyelitis.

We conducted bone puncture biopsies at the sites of bone damage in 4 children, and the findings did not indicate the presence of microorganisms. Instead, the biopsied diseased tissue exhibits fibrous tissue proliferation between the trabeculae of the bone. Lymphocytes were the predominant inflammatory cells observed in lesions, and LEM infiltration was observed in all patients. Notably, in 3 patients with CRMO, biopsy revealed the presence of both NEU and LEMs (Fig. 5).

### 3.6. Treatment

The initial management of all patients included nonsteroidal anti-inflammatory drugs (NSAIDs) as the primary treatment modality. Among the 7 patients, 6 showed favorable responses to NSAIDs, including a notable reduction in symptoms. However, 1 patient experienced a relapse after 27 months of follow-up. In response to this relapse, the patient agreed with the clinicians’ decision to initiate combination therapy with adalimumab, a tumor necrosis factor-alpha (TNF-α) inhibitor (Table 1). Subsequently, the patient experienced symptom relief.

## 4. Discussion

This single center retrospective case series sought to elucidate the demographic characteristics and clinical features of CRMO, a rare autoinflammatory bone disease with a relatively higher incidence in Europe than in other regions. This condition primarily affects adolescents and children, with an average age of onset of 11 years. Existing literature from France, Belgium, and other European countries has indicated a higher prevalence of CRMO among females, demonstrating a pronounced biological sex bias in favor of girls, as corroborated by numerous studies.^[15–17]^ Intriguingly, our study yielded contrasting observations, as the majority of CRMO diagnoses in our case series occurred in male patients. Indeed, a comprehensive review of the existing literature revealed a recurring pattern in which CRMO predominantly affects male individuals in Asian regions, including China, Japan, and India.^[18–21]^ Conversely, the United States and Chile appear to exhibit a more balanced biological sex distribution in CRMO cases.^[22,23]^ Collectively, these findings indicate that the presumed predisposition of women to CRMO is less distinct in non-European populations, indicating potential geographical and ethnic influences. This discrepancy may be attributed to several factors; the smaller sample sizes in our study may have limited the ability to capture sex-specific trends. Additionally, regional and genetic differences may influence the disease distribution, resulting in variability across populations. Although hormonal factors during adolescence may explain the higher prevalence in women, their role remains unclear, and may not be consistently observed. Differences in diagnostic criteria and referral patterns may further affect sex distribution, with some settings potentially having an increased chance of diagnosing females. It is also worth noting that CRMO has been associated with genetic factors while and a substantial body of research has identified familial clusters of CRMO involving siblings or individuals from multiple generations within a single family. Approximately 50% of individuals with CNO, a related condition, have a personal or family history of other autoimmune or inflammatory conditions, including psoriasis, inflammatory bowel disease, and inflammatory arthritis.^[24,25]^ However, consistent with the findings of several CRMO investigations conducted in China,^[18,19,26,27]^ the present study did not identify such familial clusters or family histories of CRMO. This raises the possibility of an intricate interplay between CRMO, geography, ethnicity, and genetic background, although it is essential to acknowledge that these observations may have been influenced by the relatively limited sample size in our study.

Timely diagnosis plays a pivotal role in the effective management and prognosis of CRMO. Given the unconventional symptomatology associated with this condition, the definitive identification of CRMO depends on the physician’s expertise, commonly resulting in a protracted interval between the onset of initial symptoms and formal diagnosis. An extensive study conducted in France underscored this diagnostic delay, revealing a wide range from 1 to 137 months, with an average latency period of 17.3 months.^[28]^ Notably, there has been a positive trend towards a reduction in delayed diagnoses as CRMO has garnered increasing attention and interest. In accordance with this shift, our study showed a comparatively shorter average diagnostic delay of 8.2 months, ranging from 1.5 to 24 months. This improvement in diagnostic efficiency may be attributed to the growing familiarity and expertise of medical practitioners in handling CRMO cases.

CRMO manifests as nonspecific symptoms, while some cases may be asymptomatic. Children most commonly present with localized pain as the primary symptom, which can be insidious in onset and characterized by recurrent episodes and exacerbations during nocturnal periods. Unfortunately, this pain can be misconstrued as benign growing pain, infectious osteomyelitis, or even malignant bone tumors in children,^[29]^ leading to delayed clinical intervention and undue distress in the affected child. Furthermore, localized pain tends to be most prevalent in areas such as the knees and ankles,^[29,30]^ further amplifying the risk of misdiagnosis by less experienced practitioners, who may erroneously attribute symptoms to growing pain or neoplastic conditions. Additionally, a proportion of patients may exhibit localized soft tissue swelling and limping, with physical examination revealing tenderness and increased skin temperature in the affected bones. Systemic symptoms such as fatigue and fever have been frequently reported in such cases.^[22]^ Remarkably, some patients may experience bone injury complications, such as vertebral compression fractures or deformities, even in the absence of prior symptoms.^[16]^ Without intervention, approximately 40% of cases progress to arthritis, particularly when the lesions are situated proximal to the joints.^[1]^

Although CRMO can affect almost bone in the body, the most commonly involved sites in children include the metaphyses of the long bones in the lower extremities, clavicle, mandible, and spine. Conversely, the diaphyses of long bones are rarely affected.^[14]^ Intriguingly, the skull is also infrequently implicated, with only 1 case of a CRMO lesion in the occipital bone documented in the existing literature.^[31]^ Our study found similar results, with the epiphyses of the tibia and femur emerging as the most frequently afflicted regions, characterized by an abundance of trabeculae and a higher rate of bone remodeling. The clavicle, spine, and other bones were also involved at a lower frequency. Notably, the foot bones, including the talus and calcaneus, were affected in our patient cohort. Considering the pattern of bone involvement, 1 study^[32]^ categorized CRMO into 2 distinct types. The “tibial accessory multifocal” pattern predominates, with tibial lesions featuring prominently. Approximately 65% of cases within this category display symmetric tibial lesions; over time, most of the initial focal bone involvement transitions to multifocal disease.^[28]^ The other category is the “clavicle-spine oligofocal pattern,” characterized by lesions primarily located in the clavicle without concurrent tibial involvement. In some cases, these lesions accumulate in the spine. In this pattern, the clavicle or mandible more frequently exhibits single focal asymmetric lesions that remain throughout disease’s progression.^[32]^

In our study, 1 child presented with a distinctive skin manifestation resembling SAPHO Syndrome, which was characterized by the presence of scattered pustules on the soles of the feet. However, discerning whether this condition should be classified as SAPHO syndrome or CRMO is challenging, as an ongoing debate remains regarding whether these represent distinct diseases or different facets of a broader syndrome. Typically, CRMO primarily affects children between the ages of 3.5 and 14 years, whereas SAPHO syndrome tends to affect adolescents aged between 13.5 and 17.5 years. The anterior chest wall is the most frequently affected region in SAPHO syndrome, often featuring the characteristic “bull’s head” sign observed in the sternoclavicular joints and sternum, with the spine being less commonly involved. In contrast, the spine is even less frequently affected in CRMO, while involvement of the long bones and mandible is rare.^[33]^ Given the young age at onset and site of initial involvement in our patient, we interpreted this as a manifestation of bone damage stemming from CRMO. However, it is crucial to recognize the complexity of these conditions, and the line between CRMO and SAPHO syndrome can appear blurred, warranting a nuanced evaluation and careful consideration of the clinical presentation and diagnostic criteria.

The diagnosis of CRMO poses a significant challenge, owing to the absence of specific laboratory markers. As is often the case in similar studies, 7 of our patients exhibited normal leukocyte counts in the peripheral blood, while ESR and CRP levels showed only mild elevation in 1 patient. This observation aligns with the typical clinical presentation of CRMO, as it is primarily a sterile inflammatory disease and inflammatory markers generally tend to be nonspecific, and commonly fall within the normal range, or display only slight elevations.^[34]^ Nevertheless, research has indicated that serum levels of certain inflammatory cytokines, including IL-6 and IL-12, as well as chemokines such as MCP-1, MIP-1b, and TNF-α, and eosinophil-activated chemokines, are elevated in individuals with CRMO. Moreover, there is substantial variability in the levels of soluble IL-12 receptors among patients with CRMO and healthy populations, indicating the potential utility of these cytokines as diagnostic biomarkers for CRMO.^[35,36]^ In our study, all patients underwent testing for HLA-B27, and the results were uniformly negative. This outcome is consistent with numerous investigations that have similarly reported low occurrences of HLA-B27 and antinuclear antibodies in patients with CRMO.^[25,28]^ These findings emphasize the need for comprehensive clinical evaluation and radiological assessment in conjunction with laboratory results when diagnosing CRMO, as the absence of specific markers underscores the complexity of this condition.

The neutrophil-to-lymphocyte ratio (NLR) and platelet-to-lymphocyte ratio (PLR) are emerging inflammatory biomarkers recognized for their rapid responsiveness to inflammation. These markers also offer high sensitivity but exhibit relatively low specificity.^[37]^ Over recent years, an extensive body of research has underscored the utility of NLR and PLR in various inflammatory conditions.^[38,39]^ In our study, we analyzed the changes in both NLR and PLR in individuals with CRMO. Notably, a substantial proportion of patients (42.86%) exhibited a decrease in NEU%, concomitant with an increase in the LEM%, with values ranging from 0.77 to 0.88. Compared to the NLR range observed in the pediatric population (2–18 years) in the study conducted by Azab et al,^[40]^ the NLR values in these patients were notably reduced. However, in the remaining half of the patients, NLR values ranged from 1.51 to 2.54, which is within the normal range. Notably, these ranges are subject to variations influenced by confounding factors such as race and biological sex. Furthermore, our study revealed an upward trend in platelet counts in the peripheral blood of 85.71% of patients, with PLR values ranging from 91.51 to 230.81. Regrettably, there is a paucity of studies investigating PLR values in healthy pediatric populations, making it challenging to draw direct comparisons. It is also essential to acknowledge that the statistical significance of these observed changes may be subject to debate due to the limitations stemming from the sample size in our study. Therefore, the role of NLR and PLR in monitoring the development and prognosis of CRMO warrants further investigation using larger sample sizes to provide more robust insights.

Radiological manifestations of CRMO are dynamic and evolve throughout the various stages of the disease. In the early stages of the disease, radiographs may appear normal or reveal lytic lesions. As the disease progresses, radiographs may show signs of sclerosis, bony expansion, or a mixed radiographic pattern in later stages. These atypical presentations are often misconstrued as bacterial osteomyelitis or bone tumors, contributing to diagnostic challenges.^[41,42]^ CT imaging exhibits findings similar to those of radiography, including lytic lesions, sclerosis, bony expansion, or mixed patterns. However, CT is generally less preferred in children with suspected CRMO due to concerns regarding radiation exposure. MRI is thus the preferred imaging modality for the diagnosis and monitoring CRMO. Compared to CT and X-rays, MRI has superior sensitivity for detecting the location and extent of inflammation, evaluating disease activity, and assessing bone damage.^[43]^ Whole-body MRI is particularly valuable as it provides comprehensive information on lesion distribution, soft tissue involvement, and potential organ involvement. This comprehensive insight aids in the differential diagnosis of malignancies, such as neuroblastoma. Bone marrow edema is a hallmark MRI presentation of CRMO, characterized by increased signal intensity on short T1 inversion recovery images and lower signal intensity on T1-weighted images compared to the muscle tissue.^[14,44]^ However, it is important to acknowledge that whole-body MRI (WB-MRI) may not always be feasible in clinical practice, considering economic considerations and the level of cooperation achievable with pediatric patients.

Bone biopsies serve as a valuable adjunctive diagnostic tool, particularly in cases where more serious conditions, such as malignant tumors, need to be ruled out. In CRMO, bone biopsies typically exhibit nonspecific inflammatory changes. Early lesions commonly manifest as acute inflammation, characterized by the accumulation of NEU in the bone marrow. Osteoclastic bone resorption and necrosis may also be observed at this stage. As the disease progresses, the inflammatory infiltrate tends to shift towards a composition of LEMs and plasma cells. Some patients also display granulomatous foci. In later stages, fibrosis becomes more prominent and osteoblasts become observable. There were also signs of reactive new bone formation surrounding the inflamed areas.^[4]^ Consistent with these observations, our study detected a combination of NEU, macrophages, and LEMs occupying the bone trabecular space, reinforcing the nonspecific inflammatory nature of CRMO on histological examination. This underscores the importance of considering bone biopsies in the diagnostic workup, especially when there is uncertainty surrounding the etiology of the condition or concerns regarding more serious underlying pathologies.

Treatment approaches for CRMO vary as these conditions exhibit heterogeneity in patient responses. The primary goal of managing CRMO is to alleviate pain and ensure normal bone growth, thereby maintaining regular joint function.^[30]^ Notably, standardized guidelines for the treatment of CRMO are lacking. NSAIDs are generally considered the first-line treatment for CRMO, and have demonstrated good efficacy. Wipff et al previously reported favorable responses in 73% of patients treated with various NSAIDs.^[28]^ In our study, 6 children received NSAID treatment and exhibited significant improvements in pain symptoms, which did not recur during subsequent follow-up. Steroid therapy, disease-modifying antirheumatic drugs, bisphosphonates, and biological therapies are all possible secondary treatments for CRMO when NSAIDs are ineffective. Steroid therapies, such as intravenous methylprednisolone, oral prednisolone, or hydrocortisone, often achieve initial relief, but may lead to pain recurrence upon discontinuation. The side effects associated with prolonged glucocorticoid use limit its widespread application.^[10,45]^ Traditional non-biologic disease-modifying antirheumatic drugs such as methotrexate and sulfasalazine may be ineffective as monotherapy, but can serve as adjunctive therapy in some cases.^[25,30,46]^ Bisphosphonates, particularly pamidronate, have demonstrated advantages in cases of spinal involvement by inhibiting osteoclast activity, reducing bone damage, alleviating pain, and mitigating inflammation. However, because of their potential side effects and long biological half-lives, bisphosphonates should be considered for treatment-refractory cases or patients with primary spinal involvement and structural damage.^[47–49]^

The use of biological agents in CRMO has gained prominence, particularly in cases with poor response to the aforementioned treatments. TNF-α inhibitors may be the preferred choice for CRMO treatment, particularly when co-occurring with other associated diseases such as inflammatory bowel disease and enteropathy-associated arthritis.^[50,51]^ There is also evidence to suggest a role for TNF-α in the pathogenesis of CRMO, with elevated serum concentrations in two-thirds of patients in the active phase.^[25,36]^ Furthermore, many studies have reported long-term effectiveness of TNF-α blockers in treating CRMO.^[1,28,52,53]^ Our study also indicated the benefit of TNF-α inhibitors, with 1 patient experiencing pain relief and resolution of plantar pustulosis following treatment. Nevertheless, while most cases show positive effects on symptoms and radiographic signs of activity, it is essential to acknowledge the limited experience with TNF-α blockers in CRMO in our study. Larger randomized controlled studies are necessary to compare the advantages and disadvantages of TNF-α blockers with those of other treatment modalities for CRMO.

While providing valuable insights, the present study has several limitations that could influence the applicability of our results. First, the rarity of CRMO poses a challenge in obtaining a sufficiently large sample size, as we could only include 7 patients from a single medical center over a 5-year period. This limited sample size may have introduced bias in our observations. In the future, we intend to collaborate with multiple central hospitals to access a more extensive and diverse patient pool. Additionally, the retrospective study design introduced the inherent risk of data omission, particularly regarding laboratory test results. These omissions may have affected the comprehensiveness of our analysis. Finally, the absence of genetic testing in our study precluded an in-depth exploration of the genetic underpinnings and etiology of CRMO. Future research should therefore consider integrating genetic testing data into analyses to enhance our understanding of CRMO pathogenesis and to facilitate the development of more effective preventive and treatment strategies.

## 5. Conclusion

The clinical manifestations of CRMO lack specificity, and unexplained bone pain is the most common symptom. Precise MRI examination is critical for detecting lesions, improving the diagnosis rate of CRMO, and providing essential support for early treatment. The results of this study provide useful insights into the clinical features, laboratory tests, imaging features, and treatment strategies of pediatric patients with CRMO.

## Acknowledgments

We have expressed our gratitude to the patients and their families for their participation and cooperation.

## Author contributions

**Conceptualization:** Minhua Hu, Wenxing Zeng, Jingtao Zhang, Hongsong Yan, Feng Huang, Hao Xiong, Bin Fang, Yue Li.

**Data curation:** Minhua Hu, Wenxing Zeng, Jingtao Zhang, Hongsong Yan, Feng Huang, Hao Xiong, Bin Fang, Yue Li.

**Formal analysis:** Minhua Hu, Wenxing Zeng, Jingtao Zhang, Hongsong Yan, Hao Xiong, Bin Fang, Yue Li.

**Funding acquisition:** Minhua Hu, Wenxing Zeng, Hao Xiong, Bin Fang, Yue Li.

**Investigation:** Yue Li.

**Methodology:** Minhua Hu, Feng Huang.

**Software:** Wenxing Zeng.

**Validation:** Jingtao Zhang.

**Visualization:** Yue Li.

**Writing – original draft:** Minhua Hu, Wenxing Zeng, Jingtao Zhang, Hongsong Yan, Yue Li.

**Writing – review & editing:** Feng Huang, Hao Xiong, Bin Fang, Yue Li.
